# Immediate Loading of Implants-Supported Fixed Partial Prostheses in Posterior Regions: A Systematic Review

**DOI:** 10.3390/dj13050213

**Published:** 2025-05-15

**Authors:** Giuseppe D’Albis, Marta Forte, Abdulrahman Omar Alrashadah, Lorenzo Marini, Massimo Corsalini, Andrea Pilloni, Saverio Capodiferro

**Affiliations:** 1 Department of Interdisciplinary Medicine, University of Bari “Aldo Moro”, 70121 Bari, Italy; marta.forte@uniba.it (M.F.); massimo.corsalini@uniba.it (M.C.); 2 Department of Dentistry, King Faisal University, P.O. Box 380, Al Hofuf 31982, Saudi Arabia; dr.alrashadah@gmail.com; 3 Section of Periodontics, Department of Oral and Maxillofacial Sciences, Sapienza University of Rome, 00185 Rome, Italy; lorenzo.marini@uniroma1.it (L.M.); andrea.pilloni@uniroma1.it (A.P.)

**Keywords:** immediate loading, fixed partial prosthesis, implants, prosthetic materials

## Abstract

**Background**: Modern dentistry strives to achieve increasingly less invasive procedures as the ultimate therapeutic goal. The careful selection of suitable candidates for immediate dental implants can offer an opportunity to reduce treatment time, lower the relative costs and improve overall patient satisfaction. **Methods**: A systematic search was conducted in March 2025, without any time restrictions, in Medline, Pubmed and Web of Science databases. To identify other related references, further research was performed. Articles related to current knowledge about the immediate loading of dental implants supporting fixed partial prosthesis in the posterior region were included. Articles not available in abstract form and articles not published in the English language were excluded. **Results**: A total of ten studies were eligible for inclusion in the current study. The search strategy resulted in a survival rate ranging from 86% to 100%, and a failure rate of less than 21.6%, with a mean follow-up of 55.6 months. Statistical analysis revealed no significant differences in survival rates between implants placed in the maxilla and mandible (χ^2^ = 0.42, *p* = 0.81, df = 2). Follow-up varied from one to ten years, reflecting variability both in study design and duration. **Conclusions**: The selected studies highlight the heterogeneity in immediate loading protocols for implant-supported fixed partial prosthesis in the posterior regions, emphasizing the variability in prosthetic materials and implant types, suggesting that immediate loading is a reliable, patient-centered therapeutic option with favorable long-term outcomes.

## 1. Introduction

A tendency toward less invasive procedures seems to be continuously predominant in modern dentistry [1]. This generally aims to reduce treatment durations, lower costs, minimizes postoperative pain and morbidity and improve overall patient satisfaction [2].

Moreover, in dental implantology, the process of placing a dental prosthesis on an implant immediately after surgical insertion, usually within 48 h, is referred to as immediate loading, meaning that the implant is put under functional load without waiting for a conventional healing period [3]. This approach contrasts the conventional waiting time of three months, which is used before functional loading is administered while the alveolar bone integrates with the implant [4,5]. Essentially, strict patient selection is necessary for immediate loading to provide adequate primary stability of the implant, which is defined as the mechanical stability achieved immediately after implant placement and the ideal bone conditions [6]. The principle of immediate loading aligns with the concept of minimally invasive procedures by reducing the overall number of surgical interventions and postoperative visits required. Immediate loading eliminates the need for a second surgery to place the prosthesis after implant healing, thus reducing overall surgical trauma [7]. Since the implant and prosthesis are placed in a single surgical phase, the subsequent manipulation of soft and hard tissues is minimized, reducing the risk of inflammation and postoperative complications [8]. Additionally, with immediate loading, patients can benefit from immediate functionality and esthetics, accelerating the rehabilitation process and reducing the period of discomfort and adaptation [9]. Numerous studies have been conducted to evaluate the therapeutic success of immediate loading in full-arch restorations, using between three to four implants, as well as in single-tooth restorations in the anterior region [10,11]. Immediate-loading implants have been shown to help preserve the bone and gingival architecture, preventing bone resorption and maintaining the natural gingival contour, which is crucial for optimal esthetic outcomes by integrating the principle of immediate loading into implantology practice. We adhere to the principles of minimal invasive dentistry, enhancing patient experience and optimizing clinical outcomes [12]. This article aims to assess the evidence regarding immediate loading associated with fixed partial prosthesis in posterior region. Finally, this study also assesses primary stability, complications and success rates.

## 2. Materials and Methods

### 2.1. Search Strategy

An electronic search was carried out and a thorough search strategy was created based on the PICO criteria [13]. The following is the PICO question: what is the current knowledge about immediate loading implants in the posterior region of fixed partial prosthesis? (Table 1).

Both male and female adult patients aged 18 years and older were included in the study if they received an immediate prosthetic implant placement. Thus, the selection encompassed both postsurgical implant placement immediately after tooth extraction and those which are placed with sufficient bone to insert the implants. The intervention procedure consisted of implant placements having prosthesis loaded within 0–48 h according to the immediate loading protocol. The study is also compared with an implant-supported rehabilitation that uses the conventional prosthetic loading protocol based on full osseointegration, the direct structural and functional connection between living bone and the implant surface, followed by functional loading. followed by functional loading. The study also considered prosthetic performance in terms of immediate loading, factors associated with its success and overall effectiveness.

The study protocol has been registered on the International Platform of Systematic Review Protocols. This is the registration number: INPLASY2024120112. The DOI number is 10.37766/inplasy2024-12-0112, https://inplasy.com/inplasy-2024-12-0112/ (accessed on 27 January 2025).

A literature search was conducted on PubMed and Web of Science databases to identify relevant studies related to Immediate Loading Implants with fixed partial prosthesis in posterior area. The search was performed using a combination of keywords (MeSH) terms, including: ((Immediacy [Mesh] OR (Immediate loading) OR (Immediate prosthesis) OR (Immediate function)) AND ((Implant [Mesh] OR (Dental implant) OR (Implant-supported prosthesis)).

Further manual exploration of the reference lists of all full-text articles and relevant reviews identified from the electronic search was also conducted. Additionally, manual searches were carried out in the following journals: *The International Journal of Oral & Maxillofacial Implants (JOMI), Forum Implantologicum, Clinical Implant Dentistry and Related Research, Journal of Clinical Periodontology* and *Clinical Oral Implants Research*. The inclusion criteria for the studies were defined as studies of any level of evidence, except for expert opinion. Additionally, only articles published in English and within the last 10 years were considered.

The exclusion criteria were set to letters to editors, review articles, animal studies and in vitro studies. Moreover, studies were excluded if the full text was unavailable or inaccessible. Previous review articles were not included because they did not provide additional data beyond the cited clinical cases and could have increased the risk of bias in the analysis. Two reviewers (G.D. and A.A.) performed a two-stage screening (title and abstracts first, followed by full-text) independently and in duplicate. At the second stage, a data screening and abstraction form was created to ensure study eligibility, conduct methodological quality assessments and extrapolate data on study characteristics and outcomes for the included studies. Any disagreements were handled by conversation, with the option of consulting a third reviewer (M.F.). Multiple reports from the same study were combined, making each study, rather than each report, the unit of interest in the systematic review, as stated in the Cochrane Handbook [14].

### 2.2. Quality Assessment

The quality of included studies was evaluated using appropriate quality assessment tools. Independent quality assessment was carried out by two reviewers (G.D., M.F.); in cases where evaluations were not consistent, a third reviewer (S.C.) was consulted to reach a consensus. For both retrospective and prospective studies, the Risk of Bias of Non-randomized Studies of Interventions (ROBINS-I) tool was applied, and the Cochrane risk-of-bias tool for randomized trials (RoB 2) was used for the quality assessment of Controlled Clinical Trials (CCTs) and Randomized Controlled Trials (RCTs) [15,16].

### 2.3. Data Extraction

Data extraction was conducted independently by two reviewers (G.D. and S.C.) with all primary outcomes being verified in duplicate, including 50% cross-checking of secondary outcomes, which was completed through several steps. General data extraction items included the title, authors, source and year of publication and funding information.

Secondary data were collected, elaborating on the demographic characteristics, interventions were carried out, and outcomes were registered.

In instances where numerical data were absent, the Web Plot Digitizer software 2.0 was employed to retrieve raw data in accordance with the guidelines outlined in the Cochrane Handbook. A manual extraction tool was utilized for data extraction, after which they computed average values for further analysis. Studies were excluded from the analysis if they contained missing or incomplete data, and it was not feasible to obtain clarification from the original authors.

A linear regression analysis was performed in order to assess the association of follow-up duration with the two independent categories of complications. The slope, intercept and coefficient of determination were calculated for both biological and mechanical complications. In addition, Pearson’s correlation analysis was performed in order to quantify both the strength and direction of the relationship between follow-up duration and complication rates. Presentation of data included scatter plots showing the relationship of follow-up duration with the different types of complications, where regression lines were added to help show trends.

In order to establish the relationship between categorical variables, a Chi-square test was carried out. Software was utilized to perform all the computations in order to increase the accuracy as well as the consistency of the analysis. Observational independence and sufficient expected frequencies that are over 5 were some of the conditions satisfied before the Chi-square test was performed. As part of the analysis, primary implant stability was also evaluated across the included studies. These assessments encompassed measurements such as insertion torque values, resonance frequency analysis (RFA) and Implant Stability Quotient (ISQ). The data regarding primary stability, including the specific methods and outcomes, are summarized in a dedicated table to provide a clear overview of the approaches used and their respective findings.

## 3. Results

### 3.1. Study Selection

**Figure 1:**
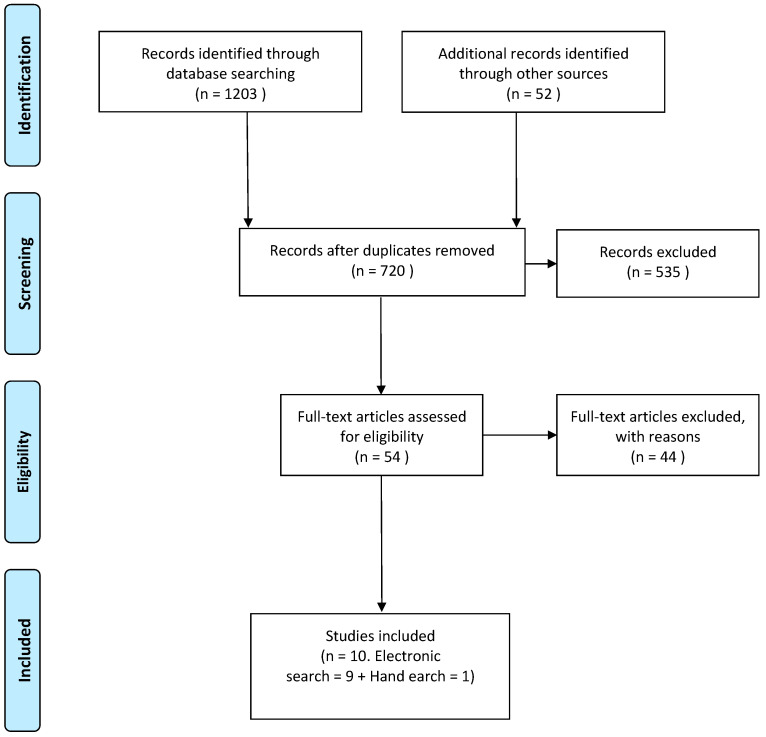
Search results, according to PRISMA 2020 statement.

The search closed on 30 December 2024. Results are presented according to the requirements of the Systematic Scoping Review Statement. A cumulative total of 1203 unique records were identified and screened by their titles and abstracts, with 52 considered relevant for full-text analysis [17]. From these, 10 of the articles met pre-set inclusion and exclusion criteria with 9 identified through the electronic search and 1 added through the hand search [18,19,20,21,22,23,24,25,26,27]. A high level of agreement was observed between the reviewers during both steps of the screening process. A total of 10 studies met the inclusion criteria for this review comprising 3 prospective studies, 2 retrospective clinical studies, 2 randomized controlled multicenter clinical trials, 2 randomized clinical trials and 1 split mouth randomized controlled trial (Figure 1).

### 3.2. Risk of Bias

Among the studies evaluated with the Cochrane Risk of Bias II tool, (Table 2) there were five studies showing some issues related to rhandomization either because of scant information or because the study design was retrospective and hence subject to selection bias. There were also problems pertaining to missing data in three studies due to inadequate reporting, particularly in studies such as those by Weerapong et al. (2019) [22] and Kim YY et al. (2021) [27]. Furthermore, in two cases, the lack of blinding of outcome assessors (Perelli M. et al., 2020, [18] and Maló P. et al., 2015 [20]) increased the risk of measurement bias even more. In general, only four studies were considered to have a low risk of bias for all domains, while the rest showed different degrees of concern, especially in studies with longer follow-up periods with high dropout rates.

The retrospective and prospective studies analyzed show varying levels of bias across the domains assessed. The cases of bias related to confounding (D1) and participant selection (D2) were mostly low in most studies, indicating a robust methodological approach in participant recruitment and control of potential confounding variables. In contrast, the classification of interventions (D3) and deviations from intended interventions (D4) posed some problems in specific studies, which may indicate possible inconsistencies in following set protocols or a lack of detailed reporting. Missing data (D5) emerged as a notable source of bias, as several studies did not adequately address incomplete datasets, potentially affecting the reliability of the outcomes. Additionally, measurement of outcomes (D6) posed some challenges, especially in studies lacking blinding of outcome assessors, leading to potential measurement bias. Lastly, bias due to selection of reported results (D7) was minimal, with most studies providing comprehensive data. In summary, while the studies generally show low to moderate risk of bias, aspects such as the reporting of missing data and consistency in intervention classification need improvement in order to enhance the reliability of the findings. These insights show how important stringent methodological practices are in both retrospective and prospective research. These findings highlight the need for increased methodological accuracy to ensure reliable findings (Table 3). None of the studies clearly stated their source of funding; however, most (6 studies) mentioned industry (implant and/or biomaterial companies) as a co-participant.

### 3.3. Data Synthesis

All the studies reported the patients as being in good systemic health; however, there was a variation in the inclusion and exclusion criteria. Some trials gave a general statement about ideal systemic health, whereas others defined certain diseases in the exclusion criteria, and a few included patients with well-controlled conditions. While the definition of periodontal status was not clearly stated, all the included trials indicated that participants had undergone periodontal therapy or were with no clinical signs of active periodontitis at the time of entry. Information about supportive periodontal care procedures and frequency was rarely reported. The implant systems and surfaces also varied. The distribution of the implants was quite wide: some studies considered only molars, others included premolars and molars. Antibiotic and postoperative regimens also differed: most of the studies mentioned that antibiotics were started on the day of surgery and were continued for 4 to 10 days, and painkillers were prescribed according to the need of the patients. The following data were extracted and organized into a comprehensive table for analysis: reference, year, number of implants, implant type, country, study design prosthesis type, number of implants lost and survival rate. The table was constructed to facilitate clear visualization and comparison of the key variables across studies, enabling detailed evaluation of the outcomes and trends. All data are summarized in Table 4.

Table 5 reports more detailed information regarding the number of implants, the length of follow-up and types of complications (both biological and mechanical). Additionally, it highlights the overall complication rate reported by the authors (%) as well as the survival rates related to the evaluated implants.

### 3.4. Incidence of Complications

**Figure 2:**
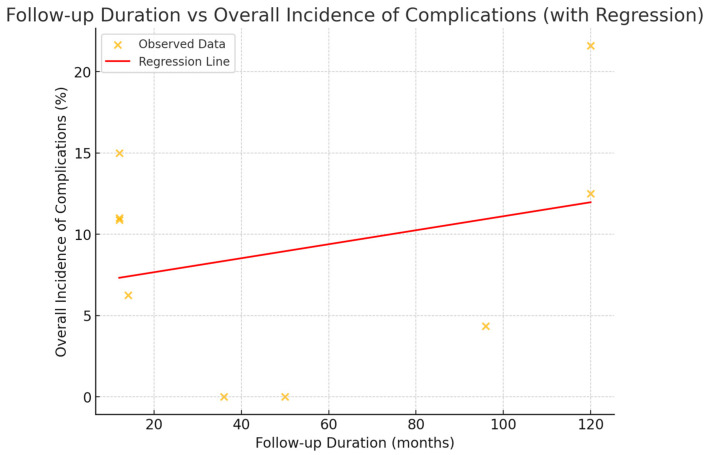
Follow-up Duration and Overall Incidence of Complications.

The results reveal a large variation in follow-up periods, ranging from 1 year to 10 years, with different rates of biological and mechanical complications. Studies with short follow-up periods, for instance one year, have reported higher rates of early implant failures and mechanical complications, such as crown fractures. On the other hand, studies with longer follow-up periods, up to ten years, reported higher rates of biological complications, for example, peri-implantitis, alongside mechanical complications including prosthetic fracture. The survival rates varied widely between studies, as some studies have reported no complications at all even after a considerable period of follow-up in the mid- to long-term, with no reportable failures. Taken together, the table allows for an appreciation of the great variability in implant performance and complication rates depending on the study design, duration of follow-up and type of implant. From the analysis of follow-up duration and overall incidence of complications, the following data were calculated (Figure 2).

A linear regression analysis revealed a weak relationship between follow-up duration and complication rates. The slope of the regression line was 0.043, indicating that for each additional month of follow-up, the complication rate increased by only 0.043%. The intercept was 6.81, the estimated rate of complication at the outset (i.e., 0 months of follow-up). The coefficient of determination (R^2^), however, was 0.081, indicating that only 8.1% of the variability in complication rates could be explained by the linear model—a sign of a very poor fit. The confidence interval was calculated to assess the reliability of the estimate, and it was determined at the 95% level.

To further evaluate the correlation, a Spearman correlation test was conducted. The correlation coefficient was 0.043 with an almost zero correlation between follow-up time and complication rate. The *p*-value was also 0.913, thereby affirming that this correlation is not statistically significant.

The data indicates that there is no meaningful relationship between follow-up duration and the incidence of complications, which could be explained by confounding variables or simply inherent variability in the data.

### 3.5. Analysis of Complications Based on Follow-Up Duration

Biological Complications:

- Slope (0.0595): For each additional month of follow-up, the rate of biological complications increases by approximately 0.0595. This indicates a gradual rise in complications over time, reflecting a steady accumulation of biological challenges.
- Intercept (−0.309): At 0 months of follow-up, the theoretical baseline level of biological complications is nearly negligible (−0.309), suggesting minimal initial complications at the start of observation.
- R^2^ (0.522): The R^2^ value of 0.522 indicates that 52.2% of the variability found in biological complications is explained by the duration of follow-up. This suggests a relationship between the duration of follow-up and the incidence of biological complications.

Mechanical Complications:

- Slope (0.1299): The mechanical complications show a steeper increase than the biological ones, with a slope of 0.1299 for each additional month of follow-up. This shows that the rate of increase in incidence with time is higher for mechanical issues.
- Intercept (−0.624): At the initial follow-up period of 0 months, the predicted baseline rate of mechanical complications is almost negligible (−0.624), showing a very low prevalence of mechanical issues at that time.
- The R^2^ value of 0.199 indicates that the follow-up duration explains only 19.9% of the variation seen in mechanical complications. This result implies that other factors, other than the follow-up duration, have significant effects on the development of mechanical complications.

**Figure 3:**
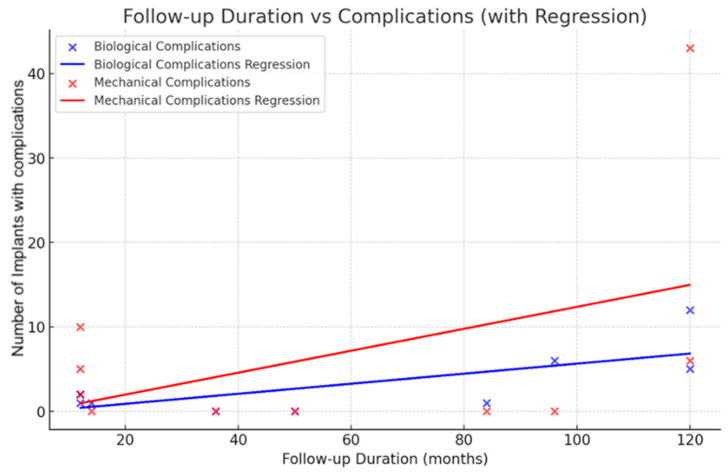
Scatter plot and linear regression illustrating the Analysis of Follow-up Duration and the Incidence of Biological and Mechanical Complications.

Biologic complications are moderately correlated with the duration of follow-up, suggesting that with increasing follow-up, biologic complications increase. The mechanical complications show a weaker association, indicating more variability or other factors not accounted for by follow-up duration alone (Figure 3).

### 3.6. Primary Stability

From the reviewed articles, the measurement of primary implant stability was frequently reported (Table 6).

All the articles emphasize that the insertion torque is a critical parameter in determining the primary stability of implants. The commonly quoted range of 30 to 50 Ncm is characterized as being essential for the successful immediate loading of implants. For instance, one study highlighted that a minimum of 35 Ncm torque should be achieved before immediate loading can be initiated. The articles describe two primary ways of evaluating implant stability.

Resonance Frequency Analysis (RFA) is used in six of the articles and is measured in Implant Stability Quotient (ISQ) units, and it indirectly reflects the stiffness of the bone–implant interface. RFA is one of the most commonly used methods for both initial and long-term evaluation of the stability of implants [23,24,25,26,27]. Manual torque wrenches were used in four studies. This is the technique of recording the insertion torque value when placing an implant. Implants are tightened to achieve the desired torque threshold for adequate mechanical stability [18,19,20,21,22]. These results show the variation in methods used in studies to evaluate primary implant stability, indicating their importance in clinical outcomes.

## 4. Discussion

Immediate loading is increasing in acceptance in order to shorten the treatment duration and improve patient satisfaction through the placement of provisional restorations shortly after implant insertion. The marginal bone levels and survival rates in immediate loading and delayed loading full-arch rehabilitations are comparable [28]. For instance, Cesaretti et al. concluded that immediate and delayed loading protocols resulted in similar survival rates and marginal bone levels over three years, thus indicating the reliability of both protocols. In another study, Amato et al. have stated that immediate loading showed cumulative success rate of 99.5%, and hence proved to be a technique that can also produce predictable results. Perilli et al. reported that the survival rates of short implants in both delayed and immediate loading conditions are comparable, therefore supporting the effectiveness of delayed protocols, most notably in avoiding the need for invasive augmentation procedures. Likewise, Daher et al., in a split-mouth study, found no significant differences in implant survival or prosthesis failure rates between immediate and delayed loading after three years, which further supports the efficacy of either approach.

On the other hand, delayed loading remains a safe alternative, even more so in cases that need longer healing periods to ensure complete osseointegration [29]. Immediate loading also has some drawbacks, such as the higher possibility of early failure, which means that the primary stability has to be strictly controlled [30].

A critical factor that can significantly influence the success of immediate loading rehabilitation is the choice of materials used for the prosthesis. Research from Maló et al. and Agliardi et al. has proven that several materials, including acrylic resin, are suitable for provisional restorations; these have been further developed into more durable alternatives such as metal-ceramic or titanium CAD-CAM constructions. These options allow for long-term function and esthetics, and they satisfy patient expectations regarding immediate function. Furthermore, the development of technology in functional CAD/CAM has considerably improved the accuracy and quality of crowns and bridges, thus increasing clinical effectiveness [31]. Amato et al. used cad-cam-fabricated hybrid ceramic crowns for immediate prosthetic treatment with further improvement of precision and efficiency in treatment. Together with Maló, Agliardi used a two-stage technique since early healing with acrylic resin bridges to be exchanged six months after insertion with titanium CAD-CAM frameworks. Only Heinemann et al. structured a touch base on the management of occlusal loading. Their trial, comparing immediate non-occluding provisional prosthesis with definitive occluding partial prosthesis, pointed out how occlusal management can weigh on functional outcomes, patient satisfaction and treatment dynamics. The focus on occlusal loading is notable in this study, as it reasserts the influence of such considerations needed for optimal results in implant-supported rehabilitations.

Graft-less procedures have become very popular in fixed oral rehabilitation protocols, as they offer a more efficient and streamlined alternative to conventional augmentation techniques [32]. A useful alternate treatment option, short implants, has emerged to be beneficial for patients with limited bone height [33,34]; this further reduces the need for invasive procedures such as sinus or ridge augmentation [35,36]. Studies by Anitua et al. and Perilli et al. have already determined that short implants have high survival rates which are not influenced by the use of either immediate or delayed loading protocols. These findings outline the ability of short implants to provide predictable results while, at the same time, minimizing patient discomfort, treatment time and costs. However, primary stability must be achieved, especially in cases of immediate loading [37,38,39].

The prosthetic retention methods, both screw-retained and cemented restorations, influence the outcomes obtained considerably. While screw-retained prosthesis offers the advantages of retrievability and ease of care, cemented options may provide superior esthetic outcomes. Research conducted by Heinemann et al. and Amato et al. demonstrates the varied applications of these retention techniques, which are customized to address particular clinical requirements.

In all the studies, the survival rates of implants and the marginal bone levels were satisfactory and high for both different loading protocols and prosthetic materials [40,41,42].

The marginal bone levels showed no significant differences between the experimental and control groups, according to the studies from Cesaretti et al. and Agliardi et al., proving once more the reliability of both methods to provide long-lasting results. Taken together, these observations emphasize the flexibility of both methods for implant prosthetic rehabilitation and their applications in different clinical scenarios [43]. The consistently good success rates achieved with most protocols and materials used are a strong testimony to the great strides made in implantology. The problems encountered, however, especially the higher immediate loading failure rates and technical complications intrinsic to some procedures, demonstrate the need for careful planning and meticulous execution to further improve clinical outcomes [44,45,46]. In recent years, particularly in the past decade, effort has been made to investigate the success rates of the immediately loaded full-arch rehabilitations, showing differences between the maxilla and mandible [47,48]. As an example, authors saw positive long-term survival outcomes for all-on-four treatment in the Japanese population sample examined, but noted a considerable difference in the cumulative survival of the maxillae and the mandible, more specifically that the maxillae had relatively worse clinical outcomes regarding the late failures, in more instances than in the mandible [49].

From the data analyzed in this systematic review, in the immediate loading in the posterior area of the jaws, the failure rates for the maxilla were comparable to failures seen in the mandible. The rates of complications reported in the various studies included in the meta-analysis ranged from 0% to 21%, and the cumulative follow-up periods for all studies varied from 12 months to 10 years. Although the statistical analysis revealed only weak correlations between follow-up duration and complication rates, it is noteworthy that studies with longer surveillance periods tended to report a higher occurrence of biological complications. However, this observation should be interpreted cautiously, as the data does not robustly confirm a moderate or strong relationship. This phenomenon appears to be consistent with results in other studies which assess the risks of biological and mechanical complications with a more traditional approach using fixed implant prosthesis subjected to loads [50]. Interestingly, Maló et al. (2015) [20] noted an increased prevalence of mechanical complications which they managed to blame on the supports and cantilevers used in their rehabilitations. It may also mean that in all the prosthetic design choices made, the ability of cantilevers to be a prosthesis design feature may lessen the possibility of mechanical complications in implant supported prosthesis [51]. In conclusion, and particularly with the two arches, immediate loading modalities seem to have an overall high survival rate, but perhaps the maxilla is more at risk of experiencing early failures [52]. Also, there is a likelihood that biological complications will increase with longer periods of follow up, leading to the strong argument for patient follow up after proper prosthetic planning and loading, so that biological complications are avoided as much as possible [53].

The analysis of the included studies showed various approaches to immediate loading protocols and prosthetic techniques. Immediate loading was often used to improve patient satisfaction and decrease treatment time. For example, Cesaretti et al. performed the immediate functional loading of implants in one hour from placement and used the octa-abutments and temporary abutments for immediate loading, while the final reconstruction was performed using metal-ceramic crowns and bridges. Likewise, Amato et al. evaluated the immediate loading of two- to four-unit fixed prosthesis in the posterior maxilla and mandible. These prostheses were first delivered as either cemented or screw-retained provisional restorations, relying upon the implant system before final abutments and impressions made afterwards, six months later. Perilli et al. immediately loaded with screw-retained provisional prosthesis 24 h postoperatively, and Anitua et al. explored immediate loading for short implants with successful outcomes, regardless of implants being splinted to another short implant or to a long one.

These findings emphasize the differences in protocols for immediate loading and prosthetic techniques while stressing the necessity of a structured approach to occlusal loading management, as emphasized in the Heinemann et al. Paper. This last aspect remains one of the crucial issues regarding the success and predictability of an implant treatment [54,55].

The interested reader will thus note, however, that many other works cited no detailed drilling protocols [56]. Out of the reviewed articles, only the work of Anitua et al. included details about the drilling technique. They employed a low-speed drilling procedure (125 rpm) with no irrigation, to reach a temperature high enough to avoid overheating of bones and allowing bone particles to be collected with grafting in mind. The use of Plasma Rich in Growth Factors (PRGF) prior to implant placement additionally characterized the method. Other works, Cesaretti et al. and Amato et al., included, as examples, some procedural recommendations for drilling protocols but did not specify these procedures, thereby causing uncertainty about such variations that could be important in outcomes. Because of the lack of these details among the majority of studies, comparisons of techniques analyzed dealt with the question of implant survival, osseointegration and complication profiles. The biological variability that this finding highlight should demonstrate the need for ongoing research to detail the methods by which the activities for implant placement are performed, particularly the drilling protocol, since these are pertinent to subsequent achieved clinical outcomes. The general identification of a protocol, guided by the review of relative success and other performance metrics, might improve consistency across such settings in the future and assist in deriving outlines for practice.

Different approaches for measuring primary stability have been employed across reviewed studies. The cited methods, which include insertion torque values, resonance frequency analysis (RFA) and Implant Stability Quotient (ISQ) measurements, are widely described in the literature [57,58]. Again, the insertion torque values were used in most studies with respect to mechanical engagement during placement. Perelli M. et al. (2020) [18] observed that the insertion torque for tapered implants (47.12 ± 6.37 Ncm) was greater in comparison to straight implants (41.60 ± 9.77 Ncm), showing the effect of implant design on stability. Thereto, Maló P. et al. (2015) [20] has defined a threshold torque value for immediate loading protocols as >30 Ncm. Other studies, Agliardi et al. (2014) [23] agreed on the threshold of ≥35 Ncm value that is sufficient to meet the stability requirement for immediate loading. Resonance frequency analysis (RFA) was again employed widely using devices such as Osstell Mentor for ISQ measures. Anitua E. et al. (2019) [19] used the RFA to monitor longitudinal stability and found that an ISQ of more than 70 predicted good stability outcomes. Manual torque wrenches were also used in studies like Weerapong K. et al. (2019) [22] to measure torque values sufficient for primary stability without a specific threshold. Some others also incorporated periodic assessments of longitudinal stability at 3, 6 and 12 months, allowing for an understanding of implant behavior in a dynamic context over time.

There are certain limitations to consider in this review. The methodologies are highly diverse and appropriate for their respective clinical and study contexts, underlining the need for standardization of their reporting for comparison across studies. The data heterogeneity taken from the included studies has proved to be a tremendous hindrance, especially regarding the radiographic evaluations [59]. Additionally, potential sources of publication bias should be acknowledged, such as the exclusion of non-English studies and the 10-year publication limit, which may have influenced the comprehensiveness of the evidence base. No extensive statistical analysis of the radiographic findings could be performed, since different authors have utilized different methodologies and reporting standards. In addition, probing data were available in only three of the studies; thus, reaching any strong conclusion about peri-implant soft tissue health was virtually impossible. The combination of data from different studies conducted in different settings and among different populations makes the differences in implant types and prosthetic designs not very well apparent. While this would help us gain a broader understanding of implant performance, this would also mask out clinically significant differences. Future research should create stratifications of these results based on the latter variables, allowing for a more delicate and personalized voicing of clinical recommendations. In addition, the limited studies presently available in the literature are long-term, and therefore, more studies need to be conducted with follow-ups to answer questions on the durability and stability of the findings in the long term. Longitudinal studies with standard reporting frameworks need to address such gaps identified by the review, as well as improve understanding of factors influencing long-term success in implants.

## 5. Conclusions

This systematic review underlines the heterogeneity of the nowadays available studies on immediate loading protocols used for implant-supported fixed partial prosthesis in the posterior regions, regarding the prosthetic materials, type and length of implants used. One feature shared by all the reviewed studies is the concept of primary stability. Despite all authors emphasizing the importance of controlling occlusal stress to avoid overloading and potential jeopardy to osseointegration, no standardized protocols for occlusal balancing are described.

The success and complication rates associated with immediate loading in posterior regions are reported at 96.5% and 8.2%, respectively, representing the average values calculated from the studies included in this review. These results suggest that immediate loading can be considered a valid therapeutic therapy, providing an efficient and patient-centered approach without compromising the integrity of long-term results. Further studies with overlapping protocols will provide additional information on the topic discussed herein.

## Figures and Tables

**Table 1:** Search strategy according to the PICO criteria.

Focused Question (PICO)		What is the Current Knowledge about Immediate Loading Implants in the Posterior Region of Fixed Partial Prosthesis?
Search	Strategy	Partially edentulous patients in the posterior regions, in the mandible or maxilla.
Population		
	
	Intervention or Exposure	Placement of implants and simultaneous positioning of the provisional prosthesis.

	
Comparison		Implant rehabilitations of partially edentulous regions without immediate prosthesis.

	
Outcome		Survival rate, complication rate.
Database Electronic search		PubMed Medline, Web of Science databases, manual search.
Journals		*Periodontology 2000, Clinical Advances in Periodontics, Dentistry Journal of Oral Pathology and Medicine, Journal of Clinical Periodontology, Frontiers in Oral Health, International Journal of Periodontics and Restorative Dentistry, Lasers in Dental Science, Journal of Periodontal Research.*
Selection criteria	Inclusion criteria	Studies at all levels of evidence, except expert opinion; articles published in English; articles published in the last 10 years.

	Exclusion criteria	Review articles, animal studies, in vitro studies. Multiple publications on the same patient population. Letters to editors. Full text not available/accessible.

**Table 2:** Risk of bias assessment based on RoB 2 for the CCT’s and RCTs included in the review.

	D1	D2	D3	D4	D5	Overall
Cesaretti G. (2015) [21]	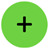	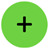	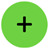	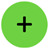	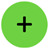	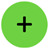
Weerapong K. et al. (2019) [22]	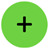	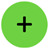	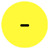	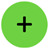	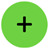	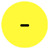
Daher FI et al. (2020) [25]	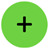	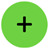	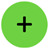	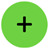	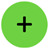	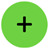
Esposito et al. (2024) [26]	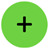	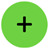	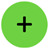	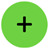	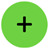	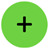
Kim YY et al. (2021) [27]	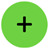	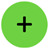	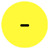	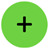	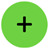	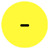

D1: Bias arising from randomization process. D2: Bias due to deviations from intended intervention. D3: Bias due to missing outcome data. D4: Bias in measurement of the outcome. D5: Bias in selection of the reported result. Judgment: 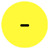 Some concerns; 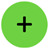 Low.

**Table 3:** Risk of bias assessment based on ROBINS I for the observational studies included in the review.

	D1	D2	D3	D4	D5	D6	D7	Overall
Perelli M. et al. (2020) [18]	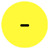	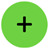	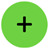	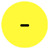	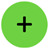	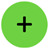	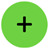	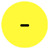
Anitua E. et al. (2019) [19]	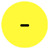	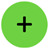	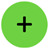	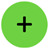	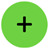	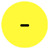	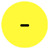	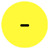
Maló P. et al. (2015) [20]	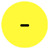	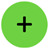	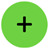	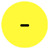	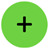	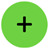	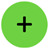	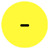
Agliardi et al. (2014) [23]	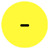	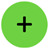	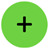	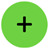	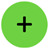	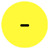	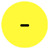	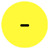
Amato F. et al. (2024) [24]	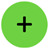	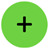	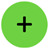	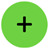	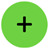	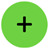	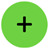	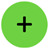

D1: Bias due to confounding. D2: Bias due to selection of participants. D3: Bias due to classification of interventions. D4: Bias due to deviations from intended interventions. D5: Bias due to missing data. D6: Bias in measurement of outcomes. D7: Bias in selection of the reported result. Judgment: 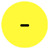 Some concerns; 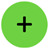 Low.

**Table 4:** Comprehensive summary of the main characteristics of the included studies.

Reference, Year, Country	Study Design	N° Implants	Implant Type	Prosthesis Haracteristics	Implant Lost
Perelli M. et al. (2020) [18] Italy	Prospective study	Max: 46 Mand: 23	Short, cylindrical, threaded implants, 7.0 mm or 8.5 mm in length.	Provisional: Acrylic resin, screw-retained. Definitive: Metal–ceramic materials screw-retained and cement-retained.	6 implants lost in maxillary region
Anitua E. et al. (2019) [19] Spain	Retrospective clinical study	48	Extra-short implants (6.5 mm) by BTI Biotechnology Institute, Vitoria, Spain.	Provisional: Acrylic resin, screw-retained. Definitive: Metal–ceramic materials, screw-retained.	-
Maló P et al. (2015) [20] Portugal	Retrospective Clinical Study	Max: 215 Mand: 266	Cylindrical, 7–15 mm length, TiUnite surface, immediate function protocol.	Provisional: Acrylic resin screw-retained Definitive: Metal–ceramic materials, screw-retained.	Maxilla: 6 lost— Mandible: 2 lost.
Cesaretti G. (2015) [21] Cuba	Randomized controlled multicenter clinical trial.	Max: 71	Straumann SLA, 4.1 mm diameter, 8–12 mm length.	Provisional: Acrylic resin, screw-retained. Definitive: Metal–ceramic materials, screw-retained.	-
Weerapong K. Et al. (2019) [22] Thailand	Randomized clinical trial	Mand: 46	PW+ Implant, 6–10 mm.	Provisional Prosthesis: Hybrid ceramic, fabricated using CAD/CAM technology, screw-retained. Definitive Prosthesis: Not specified	2 short, 1 standard implant lost.
Agliardi et al. (2014) [23] Italy	Prospective Clinical Study	Max: 20	NobelSpeedy Groovy (Nobel Biocare), axial and tilted, 11.5–25 mm length, TiUnite surface.	Provisional: Acrylic resin, screw-retained. Definitive: CAD/CAM framework with titanium and acrylic teeth, screw-retained.	-
Amato F. et al. (2024) [24] Italy	Prospective study	Max: 128 Mand: 50	-	Provisional: Acrylic resin, screw-retained. Definitive: Not specified	One implant failure.
Daher FI et al. (2020) [25] Lebanon	Split-mouth randomized controlled trial.	Max:120	NobelActive implants (Nobel Biocare), variable-thread tapered, 10–15 mm length, dual acid-etched.	Provisional: Acrylic resin, screw-retained. Definitive: Metal–ceramic materials, screw-retained.	-
Esposito et al. (2024) [26] Italy	Multicenter randomized controlled trial	72 implants: 34 occlusion, 38 non-occlusion	T3 Certain Tapered Prevail implants (ZimVie Dental), dual acid-etched, 8.5–13 mm length.	Provisional: Acrylic resin, screw-retained. Definitive: Metal–ceramic materials, screw-retained.	2 implants lost (occlusion group), 0 lost (non-occlusion group)
Kim YY et al. (2021) [27] South Korea	Randomized clinical trial	Max: 46 Mand: 56	Tapered implants (TI) (Luna, Shinhung) and Straight implants (SI) (Straumann Bone Level), SLA surface, 8–10 mm length.	Provisional: Polymethyl methacrylate (PMMA), screw-retained. Definitive: Metal–ceramic or zirconia-based materials, likely screw-retained or cement-retained.	2 TI, 7 SI implants lost

**Table 5:** The table provides a comprehensive summary of complications and follow-up data.

Reference, Year, Country	N° of Implants	Follow-Up Duration	Biological Complications	Mechanical Complications	Survival Rate %	Complications (%)
Perelli M. et al. (2020) [18] Italy	69	8 years	6 maxillary implants failed during first year	No prosthetic failures over 8 years	95.6%	4.34%
Anitua E. et al. (2019) [19] Spain	48	14 months	Higher bone loss in short-long splinted group	No prosthetic failures; distal bone loss higher	100%	6.25%
Maló P et al. (2015) [20] Portugal	481	10 years	12 biological complications; peri-implantitis	43 mechanical complications	96.7%	21.6%
Cesaretti G. (2015) [21] Cuba	71	3 years	No biological complications	No mechanical complications	100%	0%
Weerapong K. Et al. (2019) [22] Thailand	46	1 year	2 short implants failed (early failure)	Crown fractures: 3 short, 2 conventional	91.3% (short), 95.7% (standard)	10.87%
Agliardi et al. (2014) [23]. Italy	20	50 months	No biological complications	None reported	100%	0%
Amato F. et al. (2024) [24] Italy	178	6 to 10 years (mean 7 years)	1 implant failure in group 1	None reported	99.5%	0,56%
Daher FI et al. (2020) [25] Lebanon	120	12 months	Higher bone loss in second molars; peri-implantitis	Mechanical failures in prosthesis connections	100%	15%
Esposito et al. (2024) [26] Italy	72	10 years	5 peri-implantitis cases in occlusion group	Prosthetic fractures and detachment	Non-Occlusion group, 100%. Occlusion group 94.12%	12.5%
Kim YY et al. (2021) [27] South Korea	102	12 months	2 infections in GBR sites; peri-implantitis (1 case)	Prosthesis fractures (4 in TI, 6 in SI groups)	TI: 96.2%, SI: 86.0%	11%

**Table 6:** Summary of the methods and details related to Primary Stability Measurements.

Reference, Year, Country	Primary Stability Measurement
Perelli M. et al. (2020) [18] Italy	Insertion Torque: 47.12 ± 6.37 Ncm (Tapered); 41.60 ± 9.77 Ncm (Straight)
Anitua E. et al. (2019) [19] Spain	ISQ values monitored (Osstell Mentor)
Maló P et al. (2015) [20] Portugal	Insertion Torque > 30 Ncm required for immediate loading
Cesaretti G. (2015) [21] Cuba	Resonance Frequency Analysis (RFA), ISQ values > 70
Weerapong K. Et al. (2019) [22] Thailand	Manual Torque Wrenches, thresholds not reported
Agliardi et al. (2014) [23] Italy	Insertion Torque ≥ 35 Ncm (Threshold for stability)
Amato F. et al. (2024) [24] Italy	Insertion Torque recorded, values not disclosed
Daher FI et al. (2020) [25] Lebanon	ISQ measurements tracked at 3, 6, and 12 months
Esposito et al. (2024) [26] Italy	Insertion Torque: range 30–50 Ncm
Kim YY et al. (2021) [27] Republic of Korea	No explicit mention of primary stability
